# Computer-assisted diagnosis for an early identification of lung cancer in chest X rays

**DOI:** 10.1038/s41598-023-34835-z

**Published:** 2023-05-12

**Authors:** Judith Juan, Eduard Monsó, Carme Lozano, Marta Cufí, Paula Subías-Beltrán, Laura Ruiz-Dern, Xavier Rafael-Palou, Marta Andreu, Eva Castañer, Xavier Gallardo, Anna Ullastres, Carles Sans, Manel Lujàn, Carles Rubiés, Vicent Ribas-Ripoll

**Affiliations:** 1 Innovation Department, Institut d’Investigació i Innovació Parc Taulí (I3PT), Sabadell, Spain; 2 Airway Inflammation Research Group, Institut d’Investigació i Innovació Parc Taulí (I3PT), Parc Taulí 1, 08208 Sabadell, Spain; 3 Diagnostic Imaging Department, Parc Taulí Hospital Universitari, Institut d’Investigació i Innovació Parc Taulí (I3PT), Sabadell, Spain; 4 Eurecat, Centre Tecnològic de Catalunya, Barcelona, Spain; 5 Respiratory Diseases Department, Parc Taulí Hospital Universitari, Institut d’Investigació i Innovació Parc Taulí (I3PT), Sabadell, Spain; 6 Informatics and Systems Department, Granollers General Hospital, Granollers, Barcelona Spain

**Keywords:** Mathematics and computing, Cancer, Lung cancer, Radiography

## Abstract

Computer-assisted diagnosis (CAD) algorithms have shown its usefulness for the identification of pulmonary nodules in chest x-rays, but its capability to diagnose lung cancer (LC) is unknown. A CAD algorithm for the identification of pulmonary nodules was created and used on a retrospective cohort of patients with x-rays performed in 2008 and not examined by a radiologist when obtained. X-rays were sorted according to the probability of pulmonary nodule, read by a radiologist and the evolution for the following three years was assessed. The CAD algorithm sorted 20,303 x-rays and defined four subgroups with 250 images each (percentiles ≥ 98, 66, 33 and 0). Fifty-eight pulmonary nodules were identified in the ≥ 98 percentile (23,2%), while only 64 were found in lower percentiles (8,5%) (p < 0.001). A pulmonary nodule was confirmed by the radiologist in 39 out of 173 patients in the high-probability group who had follow-up information (22.5%), and in 5 of them a LC was diagnosed with a delay of 11 months (12.8%). In one quarter of the chest x-rays considered as high-probability for pulmonary nodule by a CAD algorithm, the finding is confirmed and corresponds to an undiagnosed LC in one tenth of the cases.

## Introduction

Cancer is a main cause of morbidity and mortality in the world, with near twenty million cases in 2020 diagnosed worldwide every year, being lung cancer (LC), with over two million of new cases per year, one of the most frequent tumours^1,2^. The survival of cancer patients has doubled in the last 40 years, but for LC remains low, with 1.8 million deaths due to the disease in 2020, eighty percent of the cases diagnosed that year^2^. This death rate varies substantially depending on the stage of the disease at diagnosis, but more than three quarters of the cases are detected in advanced stages, either with regional progression or metastasis, main determinants of the high mortality rate of the disease^3–5^, and an early detection of LC is considered one of the milestones to attain improvements in survival^6^.

A fundamental problem of LC is the difficulty to diagnose the disease during its initial period, due to the absence of symptoms or the non-specificity of them^7^. When a chest radiograph is performed for unrelated reasons and a LC is seen, the disease is in preclinical phase in most cases, and in this early stage it is surgically treatable and potentially curable without additional therapies. On the contrary, when the appearance of symptoms leads to the diagnosis, the disease is advanced in three quarters of the cases^8^, and requires complex treatments, with lower survival rates^5,9^.

The advances in information technology, and specifically in digital medical imaging, guarantee that radiological images are recorded and stored in digital format, often in easily reachable centralised repositories, which may be technically exploited. Even with the increase of computed tomography and magnetic resonance, simple radiology continues to be the most frequent radiologic exam, with chest x-rays as its main component. In some settings, however, up to three quarters of the chest x-rays performed for non-respiratory diseases are not informed by a radiologist and only examined by physicians from other specialties^10^. Traumatologists, internists, paediatricians or anaesthesiologists read these images, and missing a pulmonary nodule that may be an early-stage LC in this clinical situation is a possibility^11–13^. Chest x-ray is one of the most complex imaging modalities, with up to twenty percent discrepancy in their interpretation between radiologists^14^. This difficulty determines that pulmonary nodules which are early-stage LC may be missed when the chest x-ray is examined by an untrained physician, a situation that has been repeatedly reported to be related to the underdiagnosis of LC^11,12^, and has a negative impact on survival^12,13,15–20^.

Recently, methodologies based on deep learning algorithms are being successfully introduced in medical imaging^21,22^, and radiology is a prime candidate for the implementation of these techniques^23^. Computer aided diagnosis (CAD) algorithms may be integrated in diagnostic software and have been successfully used in thoracic imaging^24–26^, including chest x-ray assessment^27–30^, with accuracies over 90% for the identification of pulmonary nodules in some studies^31,32^. However, databases used until now for the creation of these algorithms have been in most cases small and with limited capabilities of machine learning^31,33–36^. Furthermore, studies that have been focused on the identification of pulmonary nodules in chest x-rays through CAD algorithms have not included a clinical validation of the radiologic diagnoses, an important point considering that part of these nodules would be malignant, and their early management before the appearance of symptoms would improve their prognosis.

The advances in information technology, and specifically in digital medical imaging, guarantee that radiological images are recorded and stored in digital format, often in easily reachable centralised repositories, which may be technically exploited. Recently developed deep learning technologies based on convolutional neural networks (CNNs)^36–39^ allow the development of algorithms capable of detecting pulmonary nodules in radiographs from data sets of medium size from these repositories, and may be continuously trained and periodically validated using additional databases^40^, and have a potential usefulness for the early detection of LC in chest x-rays performed for any reason.

The present study included the development of a CAD algorithm based on CNNs for the identification of pulmonary nodules in chest x-rays performed for non-respiratory diseases and only examined by physicians untrained in the identification of lung imaging, the subsequent validation of the algorithm result by a chest radiologist, and the clinical diagnosis of the identified nodules, identifying which of them correspond to LC, both diagnosed and undiagnosed, in a retrospective cohort.

## Results

**Figure 1:**
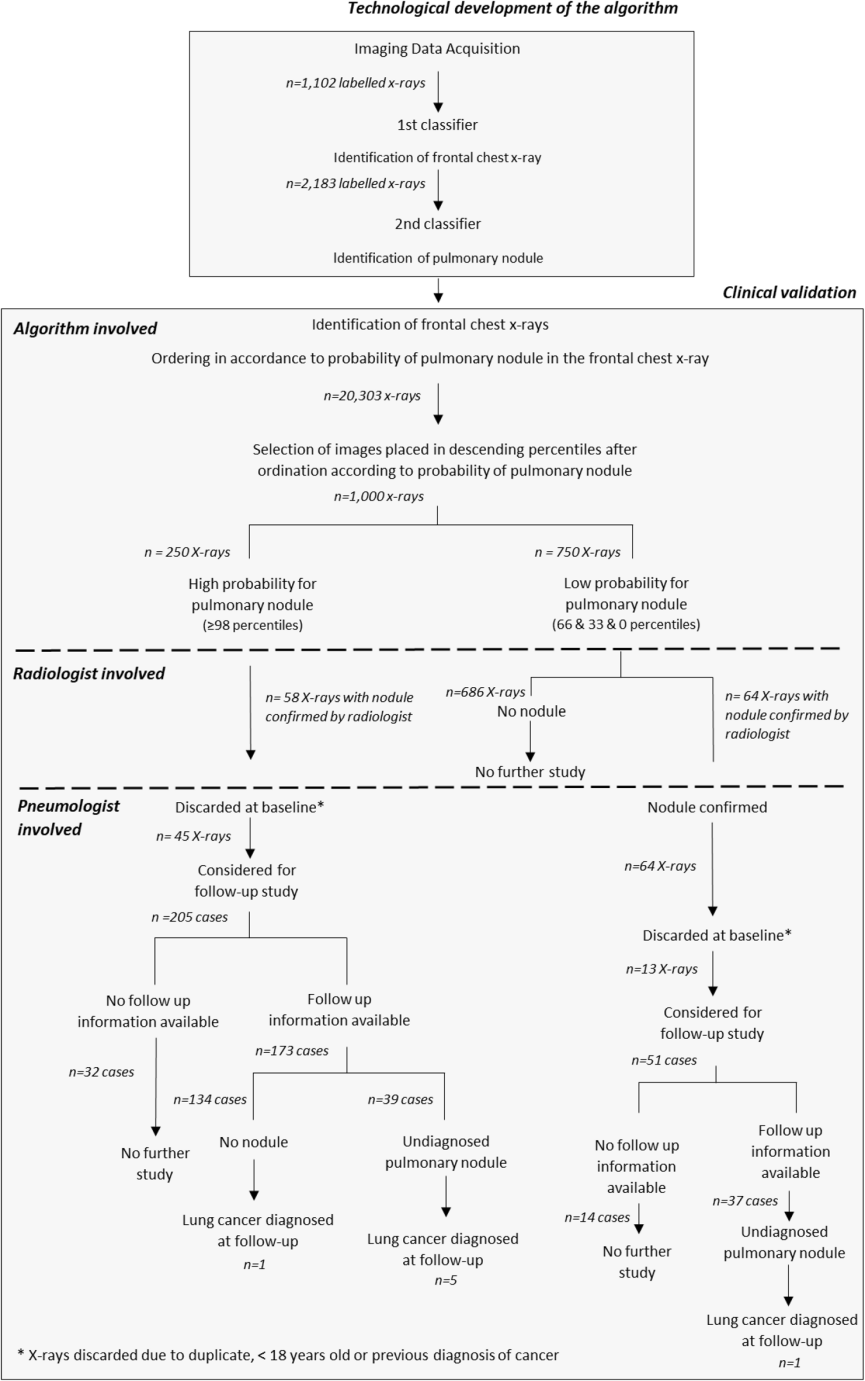
Computer-assisted diagnosis algorithm creation and clinical study.

Chest x-rays with a diagnosed pulmonary nodule and normal x-rays from the anonymised image repository of the Hospital Universitari Parc Taulí were used for the creation of a CAD algorithm using deep learning techniques and CNNs. For the preparation of the algorithm, a first classifier discerning between frontal chest images and other types of x-rays was developed, and was followed by a second classifier to differentiate x-rays with and without pulmonary nodule. The learning process first consisted in analysing 1,102 x-ray images from Hospital Universitari Parc Taulí with the first classifier, and in this stage the best model attained a sensitivity of 99% and a specificity of 95%, with an overall accuracy of 96%. The process continued using 2,183 additional x-rays, labelled according with the absence or presence of a nodule, which were also analysed, stratified and partitioned. In this second stage, the model attained a sensitivity of 0.78 and a specificity of 0.80, with an accuracy of 0.79. The algorithm was subsequently used in all x-rays performed in Hospital Universitari Parc Taulí during the first semester of 2008 (n = 20,303) which had not been examined by a radiologist when obtained, and were available from the repository (Fig. 1).

For the clinical study, the chest x-rays performed in the studied semester were sorted by the algorithm in accordance with the probability of a pulmonary nodule. Four subgroups of x-rays were defined after this sorting (percentiles ≥ 98, 66, 33 and 0), with 250 images in each subgroup, which were examined by a chest radiologist to confirm the presence of a pulmonary nodule. Fifty-eight pulmonary nodules were identified in the images ascribed by the algorithm to the ≥ 98 percentile (23.2%), while the radiologic exam only found 64 nodules in images placed in lower percentiles. The difference between the number of pulmonary nodules in the images on ≥ 98 percentile and in the three lower percentiles, when considered as a whole (8.5%), was statistically significant (p < 0.001, chi square test). Subjects who had an x-ray performed in 2008 and were assessed in the study conformed a retrospective cohort, considering the image as baseline and identifying diagnoses of LC in the following three years.

From the 250 subjects in the highest percentile (≥ 98) for pulmonary nodule according to the algorithm, 9 cases corresponded to duplicate images of the same subject and 4 were obtained in paediatric age, which were not considered for further study. Thirty-two subjects who had a known solid tumour at baseline, which was a LC in 10 cases, were also discarded for the follow-up assessment. Accordingly, the cohort in ≥ 98 percentile was reduced to 205 subjects and of these only 173 subjects (63.4%) had three-year follow-up information available and conformed the final studied cohort from this subgroup.

The presence of a pulmonary nodule at the baseline chest x-ray was confirmed by the radiologists in 39 of the 173 subjects included in ≥ 98 percentile who had not been diagnosed of a solid tumour and had follow-up information available (22.5%). In 5 subjects, the clinical exam of their follow-up records showed a diagnosis of LC a median of 11 months (range 6–36) after the baseline (12.8%) (Table 1). The follow-up of the 134 subjects placed in ≥ 98 percentile by the algorithm who did not have the presence of a pulmonary nodule confirmed by the radiologist showed a diagnosis of LC in one case, 24 months after the negative baseline x-ray (0.7%). When comparing the incidences of LC in patients with a nodule at baseline which was confirmed or discarded by the radiologist, the difference was statistically significant (p = 0.002, Fisher exact test).

**Table 1:** Cases diagnosed of lung cancer in the follow-up in the groups with high and low probability of lung cancer according to the computer-assisted diagnosis algorithm (LC = lung cancer).

Percentile	Diagnosis		
	Delay (months)	Clinical	Pathological
≥ 98	6	↑Diameter	Large cell LC + metastasis
≥ 98	9	↑Diameter	Clinical
≥ 98	36	↑Diameter	Squamous cell LC
≥ 98	11	↑Diameter	Clinical
≥ 98	15	↑Diameter	Clinical + metastasis
< 98	24	↑Diameter	Clinical

The radiologist exam of the 750 baseline chest x-rays placed in percentile categories below 98 by the algorithm identified a pulmonary nodule in 64 cases, with frequencies proportional to the descending 66, 33 and 0 percentiles (30/250 [12%], 19/250 [7.6%] and 15/250 [6%], respectively). When considering these three subgroups as a whole, two subjects in paediatric age, 5 with a LC and 6 with other solid tumours known at baseline, together with 14 subjects without available follow-up information were not considered for further analyses. Thirty-seven subjects had a pulmonary nodule identified by the radiologist in this group after the exclusion of these subjects (37/723, [5.1%]), and a case of LC was identified 8 months after baseline among them in the follow-up records (2.7%).

Accordingly, the identification of pulmonary nodules by the radiologists was significantly higher in subjects with chest x-rays considered at high probability for pulmonary nodule by the CAD algorithm. Fifteen out of 58 confirmed pulmonary nodules in patients with images placed in the high probability percentiles by the algorithm were a LC (25.9%), while from the 64 subjects in lower percentiles the image corresponded to a LC in only six cases (9.4%), a difference that was statistically significant (p = 0.016, chi square test). One third of the LC in the high probability subgroup were undiagnosed at baseline and identified with a delay of nearly one year, while this delayed diagnosis was only observed in one subject placed in lower probability percentiles.

## Discussion

In the present study we have shown that the use of a CAD algorithm created for the identification of pulmonary nodules in routine chest x-rays only reviewed by clinical physicians untrained in the interpretation of chest imaging and considered without abnormalities in the lung parenchyma is able to identify images with a high probability of pulmonary nodule, which would be confirmed later by a radiologist in near a quarter of the cases. On the other hand, nodules were only found in less than one tenth of the images placed in lower percentiles by the algorithm, a difference that was statistically significant. An undiagnosed LC was later diagnosed in more than ten percent of the high probability images with a pulmonary nodule confirmed by the radiologist, with a delay of nearly one year.

For the preparation of the CAD algorithm used in the present study, a hierarchical classifier addressed the characteristics of the image in sequential order, first discerning posteroanterior chest images from other types of x-rays, to subsequently identify which of them are at high probability for pulmonary nodule, and in these stages the attained accuracies were 96% and 79%, respectively. Previous reports on CAD algorithms used for the identification of nodules have shown similar results^29,31–33^. The usefulness of the algorithm used in the present study for the identification of pulmonary nodules was confirmed by the finding that high probability images according to the algorithm had a nodule confirmed by the radiologist in 23.2% of the cases, while images with lower probabilities only show nodules in less than ten percent of the cases, a difference that was statistically significant. Accordingly, the use of the designed CAD algorithm with a ≥ 98 percentile cutoff would allow the identification of pulmonary nodules in one quarter of the chest x-rays when revised by a radiologist, after the high probability alert raised by the algorithm.

When the CAD algorithm was used on over two thousand unreported chest x-rays during the 6-months enrolment period of the retrospective cohort assessed in the clinical part of the present study, a diagnosis of LC was confirmed for 25.9% of the images considered at high probability by the algorithm and confirmed by the radiologist. Only two thirds of these LC were known or diagnosed at baseline, while in the remaining third the diagnostic was missed, and only established one year later. Missing pulmonary nodules in the chest x-ray is a main cause of delay in the diagnosis of LC and have a demonstrated negative impact on the prognosis of the disease^15^. Quekel and cols. examined the availability of previous chest x-rays in four hundred patients with a diagnosis of non-small cell LC, and found that a pulmonary nodule with a median diameter of 16 mm was present more than one year before the diagnosis in almost twenty percent of the images, and the delay in its identification was responsible of the progression to an advanced stage in near a half of the patients^13^. Similarly, Singh and cols. examined 587 patients with LC and found missed pulmonary nodules in previously available chest x-rays as a main cause of diagnosis delay in one third of the patients, who had a LC identified half a year after the first abnormal x-ray^7^. Turkington and cols., with equivalent results in a similar population, also found that missing pulmonary nodules had a direct effect on the time to treatment, which was significantly increased in more than 100 days, and paralleled by a shortened survival in an equivalent number of days^11^. Assessing also the impact of delayed diagnosis, Sakai and cols. showed in a study on five thousand patients that the identification rate of pulmonary nodules in chest x-rays when examined by clinicians not trained in the assessment of lung images was fifty percent below trained readers, who diagnosed smaller tumours which were more often surgically-treated^12^. The use of a CAD algorithm designed for the identification of pulmonary nodules would facilitate the recognition of pulmonary nodules in x-rays read by untrained physicians, pointing to the images with the highest probability which need to be checked by a radiologist. One tenth of pulmonary nodules identified by the algorithm and confirmed by the radiologist were later diagnosed as LC in the studied retrospective cohort, and in these patients an earlier identification may have allowed an advanced diagnostic procedure and the treatment of the disease before the appearance of symptoms, with a potential improvement in survival. The identification of five LC cases during the 6 months’ period of the study confirms that the use of the CAD algorithm would have allowed an early diagnosis in up to 3% of the patients diagnosed in the region that period, considering that 200 new cases of LC are diagnosed yearly in the studied area^41^.

The present study demonstrates the usefulness of a CAD algorithm designed for the identification of pulmonary nodules in chest x-rays not examined by a radiologist. In images with a high probability for that diagnosis, considering in this category the x-rays placed in the ≥ 98 percentile by the algorithm, a pulmonary nodule was confirmed by the radiologist in near a quarter of the cases, while significant images were found by the radiologist in less than one tenth of x-rays considered at lower probability by the algorithm. A limitation of the approach followed in the present study is that the analysis of low-probability images in these x-rays was restricted to chest x-rays placed in the percentile 66 and lower, however. It is possible that the use of a cut-off between the percentiles 98 and 66 may still allow an early identification of pulmonary nodules with clinical significance. The aim of the present study was the identification of a cut-off able to attain positive identification results to support the clinical implementation of the proposed CAD algorithm in clinical practice, and has demonstrated the usefulness of the percentile 98 for this purpose. The potential usefulness of cut-offs between the percentiles 98 and 66 would need further research. The use of the proposed CAD algorithm on chest x-rays unread by a radiologist would favour the identification of patients who require fast-track access to chest computed tomography^42^, considering that this technique is the current gold standard for thoracic imaging in suspected LC. The performed research has focused in chest x-rays unread by a radiologist, and the obtained results cannot be extrapolated to chest images that include a radiology report. In this situation the added value of the used CAD algorithm is unknown and would need additional research. Accordingly, the present study supports the extended use of the CAD algorithm, and equivalent approaches able to demonstrate a similar added value, in clinical settings where chest x-rays are not read by a radiologist, to identify chest x-rays requiring a fast track access of chest CT for diagnosis.

In conclusion, a newly designed CAD algorithm focused on the identification of pulmonary nodules was able to identify high probability images in a retrospective cohort that included patients who attended a general hospital for any reason and had a chest x-ray only examined by their clinical practitioner. A pulmonary nodule was confirmed by a radiologist in more than twenty percent of these cases, and a LC missed at that baseline was later diagnosed in nearly three percent of the patients with x-rays considered at high probability by the CAD algorithm, with a delay in the diagnosis of nearly one year from the x-ray. Accordingly, the use of a CAD algorithm focused on the identification of pulmonary nodules would allow an early identification of images requiring a diagnostic procedure, which would be followed by early treatment when a LC is confirmed, improving the survival expectancy.

## Methods

### Development of the computer-assisted diagnostic algorithm

For the preparation of the CAD algorithm a hierarchical classifier was proposed decomposing the problems to be addressed in sequential order. First the classifier aimed to discern between frontal chest images and other types of x-rays, and was followed by the differentiation of chest x-rays with and without pulmonary nodules from the images labelled as frontal chest x-rays by the first classifier. The learning process analysed images from Hospital Universitari Parc Taulí with a pulmonary nodule absent or present, which were divided into train and test sets, first using 5-CV and followed by ResNet-50 for the binary classification^43,44^. The classifier was trained with a fivefold cross validation and during training the minority class was randomly oversampled to be balanced with non-nodule images. The images were resampled to 1.1 pixels and normalized to [0–1]. Data augmentation transformations were additionally used, such as rotation (degrees: − 5, 5), flip (left–right), crop padding and a resize to 320.

### Population sample

A retrospective cohort was created with all the subjects who attended the Hospital Universitari Parc Taulí in the first semester of 2008 who had a chest x-ray performed. The hospital has a reference population of 400,000 inhabitants of Vallés Occidental, part of Barcelona metropolitan region, and diagnose 200 new LC cases every year^41^. Chest x-rays performed in Hospital Universitari Parc Taulí during that period were identified, and subjects with images which had not been informed by a radiologist were selected. Baseline clinical information in hospital records was examined for every case and subjects in paediatric age or with a diagnosed solid tumour at baseline were not considered for follow-up assessment.

Participants with hospital follow-up records available for the following 36 months after baseline were identified, and the diagnosis of a LC in this period and its timing was recorded, considering a diagnosis as positive when there is an increase in the size of the pulmonary nodule compatible with the natural history of LC and/or a biopsy confirmative of that diagnosis. Obtained data were anonymised before any analysis.

### Clinical study

The trained algorithm was used on all chest x-rays performed in the population sample, and sorted them in accordance with its estimated probability of a pulmonary nodule. X-rays placed by the algorithm in the highest percentile (≥ 98/n = 250) were selected to be read by a chest radiologist^45^, for the confirmation of the diagnosis. The evolution of these patients for three subsequent years from the 2008 baseline x-ray was assessed in the hospital records, focusing on the appearance and timing of LC diagnoses. The same procedure was followed in three subgroups placed by the algorithm in descending percentiles (66, 33 and 0). Two-hundred and fifty subjects were included in each subgroup.

The clinical exam of the selected subjects included the diagnosis of any type of cancer at baseline. Their follow-up focused on the identification of LC in the subsequent three years, under the assumption that any undiagnosed LC present at baseline would progress to symptoms and diagnosis during this period, in accordance with the natural history of LC^46^. All subjects in the high percentile for a pulmonary nodule according to the algorithm were included in the follow-up assessment, independently of the report provided by the radiologist. The same procedure was followed with subjects in lower percentiles, restricted to the subjects who had a pulmonary nodule identified by the radiologist at the baseline x-ray.

### Statistical analysis

Results for categorical variables are expressed as absolute and relative frequencies and results for continuous variables as median and interquartile, when required. Sensitivities, specificities and accuracies of the created CAD algorithm in the successive stages were calculated, using the radiologist report of absence/presence of pulmonary nodule in image test sets as gold standard. In the clinical study, first the prevalence of pulmonary nodules in the assessed groups with different probabilities of that diagnosis according with the CAD algorithm were determined and compared, and, second, the incidence of new LC in the different groups was assessed, with the timing to this diagnosis from baseline, comparing the results obtained in the different groups. Finally, the prevalence of LC at baseline was determined in both groups and compared, discriminating between known and unknown LC only diagnosed later in the follow-up. Chi-square and Fisher exact tests were used for the comparisons when required. Statistical tests used were two-sided, and a p value of 0.05 or less was reported as statistically significant. Statistical analyses were performed using SPSS statistical software package version 28.0.1.0 (SPSS, IBM, Armonk, New York, USA).

### Ethics declarations

The study protocol was approved by the CLlNICAL RESEARCH AND ETHICS COMITTEE of Corporació Sanitària Parc Taulí, in Sabadell, Barcelona.

The need for informed consent was waived by CLlNICAL RESEARCH AND ETHICS COMITTEE of Corporació Sanitària Parc Taulí.

All methods were carried out in accordance with the relevant guidelines and regulations.

## Data Availability

The datasets generated during and/or analysed during the current study are available from the corresponding author on reasonable request.

## Code availability

The custom computer code/algorithms used in this study are not publically available. Notwithstanding, they could be available from the corresponding author on reasonable request.
